# Quality of Life in Mothers of Children with ADHD: A Scoping Review

**DOI:** 10.3390/children12101376

**Published:** 2025-10-12

**Authors:** Giuseppe Quatrosi, Dario Genovese, Karine Lyko-Pousson, Gabriele Tripi

**Affiliations:** 1 Department of Psychology, Educational Science and Human Movement, University of Palermo, 90128 Palermo, Italy; giuseppe.quatrosi01@unipa.it; 2 Department of Health Promotion, Mother and Child Care, Internal Medicine and Medical Specialties (PROMISE), University of Palermo, Via del Vespro, 133, 90127 Palermo, Italy; 3 Faculté de Médecine, Université de Tours, 37020 Tours, France; 4 LI2RSO, CHU d’Orléans/EPSM «Daumézon», Université d’Orléans, 45100 Orléans, France; gabriele.tripi@epsm-loiret.fr

**Keywords:** ADHD, mothers, caregivers, quality of life, well-being, parenting stress, psychosocial factors, social support, caregiver burden

## Abstract

**Highlights:**

**What are the main findings?**

- Mothers of children with ADHD typically displayed a reduced quality of life across physical, psychological, social, and environmental dimensions.
- More significant ADHD symptoms and concomitant disruptive disorders significantly exacerbate the challenges faced by mothers, particularly when they experience depression or lack of social support.

**What is the implication of the main finding?**

- Family-centered ADHD management must include periodic assessments of the mother’s health and well-being, alongside psychoeducation, coping skills training, and support groups for the child.
- Using standardized parental quality-of-life metrics and longitudinal assessments is essential to inform policy and resource allocation, such as respite care and flexible employment support.

**Abstract:**

**Background:** Attention-deficit/hyperactivity disorder (ADHD) affects not only children but also their families. Mothers, as primary caregivers, frequently experience high stress and reduced well-being. This scoping review mapped recent literature (2015–2025) on the quality of life (QoL) of mothers of children with ADHD and identified key factors influencing maternal QoL. **Methods:** Following the Arksey and O’Malley framework and Joanna Briggs Institute guidance for scoping reviews, we searched PubMed, Scopus, and ERIC in June 2025 for peer-reviewed quantitative studies in English. Eligible studies focused on mothers of children (6–18 years) with ADHD and used validated parent QoL measures. Eight studies met inclusion criteria. **Results:** Eight studies published between 2015 and 2025 satisfied the inclusion criteria. Mothers regularly indicated a worse quality of life relative to control groups, demonstrating shortcomings in physical, psychological, social, and environmental domains. Severe ADHD symptoms in children, accompanying disruptive disorders, parental distress or anxiety, and inadequate social support were important variables. Adaptive coping strategies correlated with enhanced outcomes, and a longitudinal study showed that effective ADHD intervention reduced familial stress over several months. Several studies have identified maternal depression, child comorbidities, and inadequate social support as key factors that adversely affect parental quality of life. **Conclusions:** Mothers of children with ADHD are at heightened risk for compromised QoL. Family-centered strategies that support maternal mental health, strengthen social support, and enhance coping—alongside the child’s ADHD care—are warranted. Heterogeneity in QoL measures and limited longitudinal evidence highlight priorities for future research.

## 1. Introduction

ADHD is characterized by persistent inattention, impulsivity and hyperactivity that impair the child’s functioning. It concerns approximately 5–7% of school-aged children globally [1,2,3] and frequently occurs alongside with behavioral, emotional, or learning challenges. The difficulties associated with ADHD impact not solely the child but also reverberate throughout the entire family. Parenting a child with ADHD correlates with increased stress, emotional fatigue, and caregiver burden [4,5].

Mothers often serve as the principal caregivers and care coordinators, exposing them to considerable stress in their everyday lives. A wide range of mothers with children diagnosed with ADHD report persistent fatigue, social isolation, and a sense of being overwhelmed by their child’s needs [6]. Numerous studies indicate that mothers of children with ADHD or other severe developmental disorders experience a markedly lower quality of life than mothers of typically developing children [7,8,9]. According to the World Health Organization, quality of life reflects how individuals perceive their position in life in the context of their culture and value systems and in relation to their goals, expectations and concerns [10]. As primary caregivers, mothers frequently report chronic fatigue, social isolation and an overwhelming burden, with a markedly lower quality of life compared with mothers of neurotypical children [7,8,9,11,12,13]. Recognizing and treating the quality of life of mothers in these families is becoming increasingly considered as a public health priority, not only for the well-being of the parents but also for ensuring the correct handling of the child’s condition and overall family functioning.

Although there is growing awareness of the profound impact of ADHD on family life, specific influences on maternal quality of life have not been thoroughly synthesized. Important questions remain about the extent of quality-of-life impairments and the factors that intensify or mitigate these effects. Consequently, we performed a scoping review to comprehensively map the existing information about the quality of life of mothers (or primary female caregivers) of children with ADHD. Our objectives were to: (1) describe the impact of raising a child with ADHD on maternal quality of life (QoL), including comparisons with mothers of children without ADHD; (2) ascertain the principal determinants and predictors of QoL among these mothers, encompassing child-related factors, caregiver-related factors, and broader familial or social influences; and (3) synthesize the QoL assessment instruments employed and identify any deficiencies in the research, such as methodological limitations or areas necessitating further investigation. The review aims to clarify the challenges faced by this population and assist healthcare providers and policymakers in determining the allocation of supportive resources. Enhancing mothers’ quality of life may consequently yield beneficial benefits on the well-being and developmental outcomes of children with ADHD.

## 2. Materials and Methods

We followed a scoping review methodology to broadly explore and summarize this emerging area of research [14,15]. We adhered to the framework established by Arksey and O’Malley (2005), which encompasses determining a specific scope and research questions, conducting a comprehensive literature search, selecting relevant studies, charting the data, and collating and reporting the results [16]. We also consulted the Joanna Briggs Institute (JBI) guidance for scoping reviews and adhered to the Preferred Reporting Items for Systematic Reviews and Meta-Analyses extension for Scoping Reviews (PRISMA-ScR) for transparent reporting [17,18,19].

### 2.1. Research Questions

This review was guided by two primary research questions:

1. How does having a child with ADHD affect the quality of life of mothers (or primary female caregivers) compared to mothers of children without ADHD?
2. What factors (child-related, parent-related, and contextual) influence or predict the quality of life of these mothers?

Through these questions, we sought to capture both the overall impact of caring for a child with ADHD on maternal QoL and the specific determinants that might exacerbate or mitigate that impact.

### 2.2. Search Strategy

A comprehensive literature review was performed across three electronic databases: PubMed, Scopus, and ERIC. No protocol was registered. The search was conducted in June 2025 to guarantee relevance. The research group selected search terms that combined concepts of ADHD, quality of life, and parents/mothers, adopting filters for publication years ranging from 2015 to 2025. The following search string was finally used for the study purpose:

(“Attention Deficit Disorder with Hyperactivit*” OR ADHD) AND (“quality of life*”) AND (“mother*” OR “parent*” OR “caregiver*”) AND (“adolescent*” OR “child*”).

### 2.3. Study Selection

All retrieved references were exported to a reference manager (Zotero 7.0.16 (64-bit), Fairfax, Virginia, USA), and duplicates were removed. Titles and abstracts of the remaining unique records were screened against predefined inclusion criteria. We included peer-reviewed empirical studies (of any quantitative design, excluding qualitative-only studies) that met the following criteria:

- Population: It comprised mothers or primary female caregivers of children/adolescents (approximately 6–18 years old) with a diagnosis of ADHD. We excluded studies that did not specifically report results for mothers or that focused on broader family members without isolating results regarding the mothers of children with ADHD.
- Concept: The study quantitatively assessed the quality of life of the parent, using a validated QoL instrument or a quantitative measure of well-being/health-related QoL. Studies that only examined child outcomes or unrelated concepts (e.g., parenting practices or child treatment efficacy) were excluded.
- Context: Any country/setting was eligible. We included both community and clinical samples. We excluded non-peer-reviewed literature (theses, conference abstracts) and other review articles or opinion papers.

**Figure 1:**
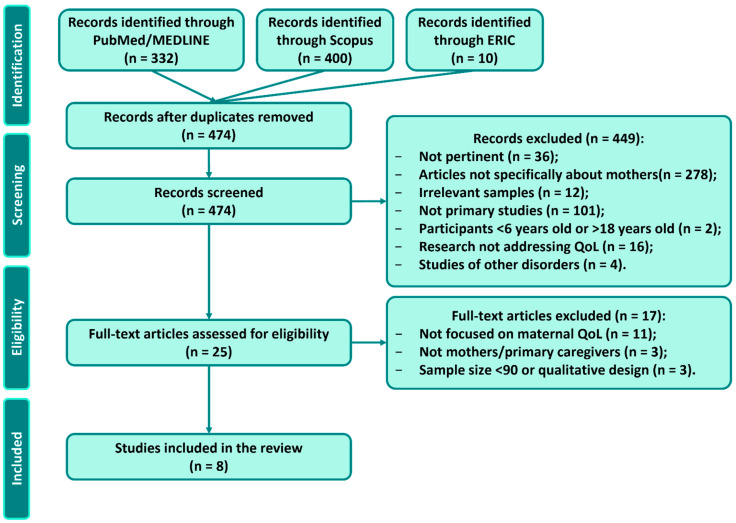
Study selection flow diagram (PRISMA-ScR).

At the screening stage, if relevance was uncertain from the abstract, the reference was retained for full-text review. Full-text articles were then obtained and assessed in detail by two reviewers against all criteria. Reasons for exclusion at full-text were documented (e.g., did not measure parent QoL, not focused on ADHD, qualitative design, and sample size too small). As an additional eligibility criterion to enhance methodological robustness, we required studies to have a sample size of at least 90 participants (this threshold was chosen to exclude very small-sample studies that might have limited generalizability or statistical power). Any ambiguities in selection were discussed with a second reviewer (senior author) until consensus was reached. Figure 1 presents the selection flow diagram. In total, out of 474 unique records initially identified, 25 articles were reviewed in full, 17 were excluded, and 8 met all criteria. One more study was added via reference mining, yielding 8 included studies.

### 2.4. Data Charting and Extraction

We developed a data-charting form to extract key information from each included study. The following data items were collected, i.e., citation details (first author, year), country of study, study design, sample characteristics (sample size of mothers, child age range, etc.), whether a comparison group was included, the QoL measurement tool(s) used, and the main outcomes/findings related to maternal QoL. To help with the comparison of studies, the results have been organized into a summary of the principal characteristics of the included research, together with the quality-of-life measures and key outcomes for each study.

### 2.5. Quality Appraisal of Included Studies

We conducted a quality assessment of the studies included to inform our synthesis (though no study was excluded based on quality). Methodological and reporting quality were appraised using the Quality Assessment with Diverse Studies (QuADS) tool [20]. QuADS is a 13-item tool developed for systematic reviews that include heterogeneous study designs. Each criterion is assigned four points on a scale between 0 and 3 (clear aims, appropriate methodologies, robust data analysis, generalizability, etc.), resulting in a maximum attainable score of 39. Two reviewers independently evaluated each work with the QuADS criteria, and any discrepancies were discussed. The QuADS tool has demonstrated strong inter-rater reliability and content validity in appraising mixed-method and observational studies [20]. We used the quality assessment to identify common strengths and limitations in the literature, which are described in the Supplementary File S1.

### 2.6. Collating, Summarizing, and Reporting the Results

We synthesized the findings narratively, in accordance with our scoping review objectives and questions. Initially, we provide an exhaustive overview of the included studies, detailing study designs, geographical areas, sample sizes, and tools for measuring QoL. Subsequently, we define the significant findings regarding the impact of raising a child with ADHD on a mother’s quality of life and the variables influencing this QoL. We grouped these findings into child-related factors and mother/parent-related factors (including family/social context) for clarity. Finally, in the Discussion, we interpret these results in the context of broader literature and discuss implications for clinical practice and future research.

## 3. Results

### 3.1. Characteristics of Included Studies

Eight studies were included in this scoping review, published between 2015 and 2024 (Table 1). They were conducted across diverse regions: two in Europe (one in France [21] and one in the United Kingdom [22]), three in the Middle East (including one in Saudi Arabia [12] and one in Egypt [13]; the third was a collaborative study in the Middle East region [9]), one in Asia (Taiwan [7]), one in North America (United States [8]), and one multi-center study in Spain (which we classified under Europe) [23]. All included studies had quantitative designs with relatively large samples (per our inclusion criteria of *n* ≥ 90 mothers or caregivers). Five were cross-sectional observational studies, and two incorporated comparison groups of mothers of children without ADHD [9,22]. One study was a prospective observational follow-up without a separate control group [23].

Table 1 summarizes the main characteristics of the eight included studies, including design, sample size and presence of control groups [12,13,21], though some also included community samples or national surveys [8,22]. Across the included studies, children’s ages ranged from approximately 6 to 18 years and, where indicated, some studies included a comparison group of mothers of neurotypical children; further details are provided in Table 1.

### 3.2. QoL Measures and Key Outcomes

Across the eight studies, maternal well-being was assessed using a range of validated instruments, including WHOQOL-BREF, EQ-5D, the parental Quality of Life scale (QoL-P) and the Family Strain Index (see Table 2) [7,9,12,13,21,22,23,24,25,26,27,28].

Despite the heterogeneous measures, all were validated instruments addressing aspects of caregiver well-being. Several studies included supplemental measures such as: parental depression (e.g., CES-D, the Center for Epidemiologic Studies Depression Scale) [8,29], parental ADHD symptoms (Adult ADHD Self-Report Scale) [22,30], parenting stress indices, and social support/family functioning scales (e.g., the Family APGAR used in Liang et al. and Azazy et al.) [7,13,31,32]. These were used to analyze predictors of QoL. Notably, through the use of established tools, each study guaranteed that the QoL outcomes were reliable and could be compared with recognized benchmarks. For example, Liang et al. confirmed that the WHOQOL-BREF could distinguish the ADHD vs. control groups, validating its use in that context [7].

Main QoL outcomes: despite differences in measures, studies have consistently shown that mothers of children with ADHD experience markedly lower levels of life compared to control groups across multiple domains. In Liang et al.’s Taiwanese sample, mothers of ADHD children had worse WHOQOL-BREF scores in all four domains (physical, psychological, social, environment) compared to mothers of non-ADHD children [7]. Approximately 67% of women with ADHD in the study were assessed as having a poor overall quality of life, in contrast to roughly 30% of mothers in the control group who were rated similarly, indicating a significant difference [7]. Cappe et al. observed that parents of children with ADHD exhibited the lowest scores on the psychological well-being and daily life activities QoL subscales, indicating significant distress and struggles with their daily lives [27].

In quantitative terms, health-related QoL utility scores were markedly lower for mothers of ADHD children. Piscitello et al. found an average EQ-5D index around 0.73 in the ADHD mother group versus 0.93 in the comparison group (on a scale where 1.0 = perfect health) [8]. This translates to a substantial deficit in quality-adjusted life years; they estimated that raising a child with ADHD was associated with a loss of about 1.96 QALYs for the mother over the child’s youth (ages 5–18) [8]. Peasgood et al. likewise observed significant decrements in mothers’ EQ-5D scores, as well as lower life satisfaction and poorer mental well-being (measured by the Warwick-Edinburgh Mental Well-being Scale) in the ADHD group [22]. However, interestingly, Peasgood et al. noted no significant difference in self-rated general health or satisfaction with income, suggesting that the burden manifests more in mental, social, and daily-life domains rather than physical health or financial perception (perhaps because some families receive resources/support) [22]. Contrariwise, other studies (such as Liang et al. and Azazy et al.) revealed a correlation between reduced household income and diminished maternal quality of life, indicating that financial hardship may intensify the challenges faced by mothers [7,13].

Each study evaluating parental stress indicated that it is markedly elevated in mothers of children with ADHD. For instance, Ahmed et al. reported that parenting stress levels were dramatically elevated in the ADHD group compared to controls [9]. Mothers often reported feeling overwhelmed: common issues included fatigue due to managing the child’s behaviors and sleep disturbances caused by the child (some mothers slept poorly because of the child’s nighttime problems or due to constant worry) [22]. Social life and relationships were also adversely affected. Many mothers had reduced time for leisure or personal activities; Peasgood et al. quantified this, finding significantly lower satisfaction with leisure time among ADHD caregivers [22]. Some studies (e.g., Cappe et al.) noted strains on the couple/marital relationship and family cohesion as well, with low spousal support correlating with worse maternal outcomes [27].

Several specific symptoms and co-occurring problems in the child were linked to even poorer maternal QoL. If the child had severe hyperactivity, frequent oppositional behaviors, or comorbid conditions (like autism spectrum traits or conduct disorders), the mother’s QoL was further reduced [7,9]. For example, one analysis found that mothers of ADHD children with co-occurring oppositional defiant disorder reported the lowest QoL, indicating the compounding burden of dealing with more challenging behavior (this finding was noted in discussion across studies). Child emotional problems (anxiety/depression) were also associated with worse maternal QoL in some reports. Interestingly, in the Taiwanese study, the child’s age had an effect: mothers of adolescents with ADHD had slightly lower QoL than those of younger children, possibly because adolescent issues (academic pressures, risky behaviors, transition to adulthood) add new stressors for parents [8]. Despite heterogeneity in measurement tools, the findings converge on three core factors associated with lower maternal QoL: greater severity of ADHD symptoms and comorbidities (particularly oppositional or autistic traits), the presence of maternal depression or anxiety, and limited social support networks.

## 4. Discussion

### 4.1. Impact of Child ADHD on Maternal Quality of Life

The results of this scoping review reveal that parenting a child with ADHD can significantly reduce a mother’s quality of life. Indeed, m others of children with ADHD consistently reported a lower quality of life than the counterparts of non-ADHD children, across different countries and measurement approaches. This finding aligns with prior literature showing the far-reaching toll of chronic child behavior problems on caregiver well-being [33,34,35,36]. In practical terms, the magnitude of QoL impairment observed is striking. Several studies suggest that a majority of these mothers experience a level of life quality that would be considered poor or unsatisfactory [7,9]. For example, two-thirds of mothers in one sample rated their QoL as poor, which is a dramatically higher proportion than seen in general population surveys [7]. Converging cross-sectional analysis reveals that parents of children with ADHD report significantly lower scores on WHOQOL domains compared to community norms, displaying significant drops in parental well-being [34]. Socioeconomic analyses further reveal higher mental health-related healthcare use and indirect costs for mothers of children with ADHD, highlighting the considerable burden beyond psychological distress [37].

The psychological and social domains of QoL appear to be the most adversely affected. Mothers frequently experience higher stress, anxiety, and symptoms of depression [9,22]. The constant challenges of managing ADHD-related behaviors—such as impulsivity, defiance, or hyperactivity—can erode a mother’s mental health over time. Many mothers report feeling emotionally exhausted and overwhelmed, which corresponds to lower scores on mental well-being indices (e.g., in the UK study, ADHD caregivers’ mental well-being scores were significantly below population norms) [22]. Socially, these mothers frequently face isolation. They may withdraw from social activities due to their child’s difficulties in public settings or due to embarrassment and stigma, as noted in the Saudi study where perceived stigma was a theme associated with lower QoL [12]. Additionally, their relationships—both within the family and externally—can become strained. Marital relationship strain is commonly reported; indeed, previous research has found higher divorce rates among parents of children with ADHD than among other parents [38]. Although our review did not specifically quantify divorce or marital satisfaction, some studies, such as Cappe et al., indicate that spousal support substantially affects maternal QoL [27]. When that support is lacking, the burden on the mother intensifies, contributing to worse QoL. Prospective cohort data indicate that parents of children with ADHD are considerably more prone to divorce and separate earlier than those in the control group, underscoring the relational stress linked to persistent externalizing symptoms [38]. Consistently, clinical samples document reduced marital satisfaction among parents of children with ADHD versus controls [39].

It is also important to highlight the physical and daily-life impact on these mothers. While some studies found physical health per se (e.g., self-rated health status) did not differ drastically from controls [22], there are indirect physical consequences. Sleep disruption is a notable issue: mothers often get less sleep or poorer quality sleep because of their child’s nighttime problems or because of their own worry and vigilance [22]. Chronic sleep deprivation can impair daily activities, reduce energy levels, and adversely affect the immune system. Mothers of children with ADHD experience fewer opportunities for self-care, exercise, or medical consultations, thereby jeopardizing their long-term health. One could interpret the lower “environmental” domain scores (seen in WHOQOL-BREF results) as reflecting financial strains and safety/comfort issues in daily life stemming from the child’s condition [7]. For instance, some mothers reduce work hours or stop working to manage their child’s needs, resulting in economic pressure. Interestingly, a major study conducted in the UK found no disparity in economic satisfaction between families with ADHD and control families [22]. This may be partly due to some families accessing disability benefits or employing adaptive strategies. Still, other studies (such as Liang et al. and Azazy et al.) did find that lower household income correlated with worse maternal QoL [7,22], suggesting that financial hardship can exacerbate the burden. Pediatric sleep studies linked ADHD-related sleep difficulties with diminished caregiver mental health and family dynamics, while randomized trials demonstrate that behavioral sleep interventions could improve parental mental well-being [40,41].

Child age is another factor to consider. One might assume that managing a young child with ADHD is hardest (due to high energy and need for constant supervision), but our review suggests that the strain does not necessarily diminish as children grow into adolescence. In fact, older age of the child was modestly associated with worse maternal QoL in at least one study (Piscitello et al.) [8]. Adolescence may introduce new challenges, including academic pressures, more autonomy (along with potential risk-taking or school-related challenges), and the impending transition to adulthood, all of which can contribute additional stressors for mothers. The needs of a teenager with ADHD (e.g., coordinating treatment as the child seeks more independence, concerns about driving safety, preparing for college or employment) can be as taxing as those of a younger child, just in different ways. Consequently, the burden on mothers may endure or even escalate during the adolescent years, rather than naturally declining.

Overall, parenting a child with ADHD constitutes a considerable chronic stressor for mothers, affecting many aspects of life. The notion of a “ripple effect” is apt—the child’s ADHD symptoms ripple outward, affecting the mother’s mental health, her relationships, her daily routines, and overall life satisfaction [12]. The public health implication is that mothers in these families represent a vulnerable group whose needs often go under-addressed in standard ADHD care (which tends to focus on the child). Our review underscores that addressing maternal QoL is not only important for the mother’s sake but likely beneficial for the child and family as a whole, since a less stressed, healthier parent can better support the child’s treatment and development. A meta-analytic study indicates that parents of children with mental disorders, including ADHD, experience substantial declines in quality of life compared to parents of healthy children, thereby positioning maternal ADHD caregiving within the broader framework of caregiver burden literature [42].

### 4.2. Factors Influencing Maternal Quality of Life

Our findings reveal the factors that significantly impact maternal quality of life when parenting a child with ADHD, including child characteristics, maternal traits, and broader familial and social factors. Understanding these factors may facilitate a concentrated effort on solutions to the most urgent areas of need.

Perhaps, unsurprisingly, the severity of the child’s ADHD symptoms emerged as a core determinant of maternal QoL in multiple studies [7,27]. When a child’s ADHD is more severe—for example, characterized by extreme hyperactivity, constant impulsiveness, or inattention that causes major daily dysfunction—the caregiving demands intensify. Mothers of these children typically have more struggles at home and school, require additional supervision, and experience more conflicts, such as those related to homework or inappropriate conduct in public. This directly translates into higher stress and less personal time, thereby lowering QoL. Some evidence also indicated that symptom type (inattentive vs. hyperactive–impulsive) might play a role: Liang et al. found that the child’s inattentive symptoms in particular were linked to reduced maternal QoL [7]. This could be because inattention often manifests in academic underperformance and organizational problems, which can be a chronic source of stress (e.g., mothers spending hours each evening helping with schoolwork or managing the child’s forgetfulness and disorganization). Complementary evidence indicates that children’s inattentive and oppositional symptoms, together with maternal mood symptoms and lower family support, are associated with poorer maternal QoL [35].

Comorbid behavioral disorders, especially oppositional defiant disorder (ODD) or conduct problems, were highlighted as significant risk factors for maternal distress [7]. A child who is not only inattentive/hyperactive but also openly defiant or aggressive creates a particularly challenging home environment. Several studies noted that mothers of children with ADHD + ODD had among the lowest QoL scores in their samples. Consistent with this, child emotional disorders (like anxiety or depression) also negatively affected mothers’ QoL, though these may manifest more as worry and the need for increased emotional support, rather than external conflict. Liang et al. observed that autistic traits in the child predicted lower maternal QoL as well [7], reinforcing that any additional developmental disorder amplifies the burden on the caregiver. Notably, offspring oppositional-defiant/conduct problems also predict earlier marital dissolution in ADHD-affected families, evidencing systemic strain beyond the mother–child dyad [38].

Another child factor was age. As previously mentioned, parenting an adolescent with ADHD can introduce additional challenges. Piscitello et al. discovered that mothers of adolescents with ADHD exhibited a marginally reduced quality of life (in terms of health utility) compared to mothers of younger children, even after controlling for additional factors [8]. Adolescence can introduce issues such as the teens’ emerging need for independence, potential for risky behaviors, and transitional planning (e.g., preparing for college or vocational training), which may cause mothers significant anxiety and require increased oversight during a period when most parents expect to grant their child more autonomy. Thus, interventions may need to support mothers through the adolescent phase as actively as in early childhood.

Among maternal characteristics, mental health status stood out as a critical influence on QoL. Mothers who had higher levels of depression or anxiety consistently reported lower QoL [7,8]. In some analyses, maternal depression was the strongest predictor of QoL, even more so than child ADHD severity [7]. This highlights that the psychological well-being of a mother is intricately connected to her perception of quality of life. The child’s condition can create a detrimental cycle: maternal depression and stress may arise, subsequently affecting the mother’s perception of life quality and her ability to cope. Identifying and treating maternal mental health issues (e.g., through counseling or medication for the mother if needed) might therefore yield significant improvements in QoL.

Maternal ADHD symptoms themselves are another important factor. Peasgood et al. noted that mothers who screened positive for adult ADHD had worse QoL outcomes [22]. ADHD frequently occurs in familial settings, and a mother with ADHD may struggle with organization, consistency, and emotional regulation, which might exacerbate the challenges of caregiving. This suggests that assessing mothers for ADHD (and providing appropriate support or treatment for the mother if ADHD is present) could be an important component of family care in these cases. Maternal ADHD symptoms are linked to higher parenting stress and less positive parenting, suggesting assessment and support for maternal ADHD may indirectly protect QoL [43]. A systematic review also indicates that parents of children with ADHD tend to rely more on avoidant-focused coping, whereas problem-focused strategies are associated with better adjustment—an actionable target for intervention [44].

Coping style was frequently mentioned as well. Cappe et al. found that mothers who relied on emotion-focused or maladaptive coping (such as denial, behavioral disengagement, or self-blame) had poorer QoL [27]. In contrast, problem-focused coping strategies and a stronger sense of control or self-efficacy were associated with better QoL for mothers [27]. This suggests that interventions aimed at training women in successful coping and stress-management strategies, such as cognitive–behavioral methods for reframing negative thoughts or systematic problem-solving skills, may mitigate certain dimensions of quality-of-life impacts.

Social support has proven to be a crucial protective element for maternal quality of life. Mothers with robust support networks, such as husbands, extended family, friends, or support organizations, typically experienced an enhanced QoL [7,27]. Conversely, reduced perceived social support and familial dysfunction, such as inadequate family communication or cohesiveness, were associated with a reduced QoL [7,13]. Conversely, low perceived social support and family dysfunction (e.g., poor family communication or cohesion) were tied to worse. Single mothers of children with ADHD were often identified as a particularly vulnerable subgroup; lacking a partner’s support can intensify both the practical workload and the emotional burden on the mother [7,22]. In ADHD samples, greater perceived social support is inversely associated with parenting stress, consistent with its role as a protective factor for caregiver well-being [45].

Economic factors were also relevant. Although not all studies found income effects (as noted, one UK sample did not see differences in income satisfaction [22]), others did observe that families with lower socioeconomic status had mothers with lower QoL [7,13]. Financial strain can add to stress, limit access to resources (like paying for extra tutoring or childcare), and compound feelings of inadequacy or worry about the child’s future It is important to point out that some countries offer subsidies or services to families of children with disabilities, potentially mitigating these challenges; differences in study outcomes may partially stem from disparities in social welfare systems. A study on economic burden indicates that ADHD increases maternal healthcare costs and reduces productivity within households [37].

Alenezi et al. explicitly investigated cultural context and stigma in Saudi Arabia, aspects often neglected in other investigations [12]. In certain cultures, bearing a child with a behavioral disorder may result in stigma for the family, leading mothers to experience blame or social ostracism. Alenezi et al. reported that mothers experiencing elevated stigma associated with their child’s ADHD experienced a lower quality of life [12]. This indicates that public perceptions and cultural acceptance of ADHD may indirectly affect mother well-being. It underlines the significance of public education and stigma reduction to support these families.

It is important to recognize that these factors do not act in isolation. The severity of the child’s symptoms may exacerbate maternal depression and strain social support, whereas effective coping skills can mitigate these effects. Several included studies performed multivariate analyses that indicated maternal mental health and social support often explained as much variance in QoL as the child’s clinical characteristics [7,22]. This is an important insight: it means that even if the child’s ADHD cannot be “cured,” improving the mother’s mental health or support network could significantly improve her QoL. Mediation studies reveal that parental stress and self-efficacy partially explain the relationship between ADHD status and reduced marital satisfaction, emphasizing variable caregiver factors [46].

These findings indicate potential targets for intervention. Parent training programs can address child behavior struggles such as rebellion or routine structure, while therapy or support groups for mothers can assist with maternal challenges like depression or coping strategies and provide social support. Socioeconomic support, such as respite care and flexible employment options, can mitigate financial and daily challenges. Randomized trials indicate that psychoeducation and behavioral parent training improve family results, while sleep-focused therapies promote parental mental health—practical approaches to elevate maternal QoL [41,47].

### 4.3. Implications for Research and Practice

The evidence discussed in this review has several implications.

First, it underscores the necessity for healthcare providers to consider the family unit, particularly the mother, as an essential part of ADHD management. Pediatricians, child psychiatrists, and psychologists must remain on alert for signs of caregiver stress and should promptly refer mothers for needed assistance, such mental health counseling or parenting training programs. Periodic ADHD clinical visits might include brief assessments to evaluate parental stress or depression. Given the correlation between maternal mood disorders and diminished quality of life in children with ADHD, it is essential to incorporate routine screening and referral mechanisms to identify depressed or anxious mothers within pediatric ADHD programs [8].

Building on the evidence summarized above, several practical interventions have shown promise in supporting mothers caring for children with ADHD. In particular, the following evidence-based strategies deserve consideration:

- Psychoeducation and parent training: structured programs combining information about ADHD, behavioral management and coping techniques have been shown to reduce parental stress and improve quality of life.
- Support groups: participation in peer support groups offers opportunities for sharing experiences and reduces social isolation.
- Cognitive–behavioral techniques: interventions focused on stress management and cognitive restructuring can alleviate maternal depressive symptoms and enhance well-being.
- Sleep and physical health interventions: improving the child’s sleep can have positive spill-over effects on maternal mental health.

The findings advocate for the setting up of family-oriented services by governmental and healthcare institutions. This may include clinics offering multiple areas of expertise, where, for instance, the mother can attend a stress management or support group session while the child receives behavioral therapy for ADHD. School systems should consider interventions targeting parents, as mothers encountering struggles with their quality of life might find it difficult to adhere to the behavioral goals for their children both at home and in school. Policy models should consider caregiver-directed components (e.g., structured parenting programs, sleep support), which have demonstrated benefits for parent outcomes in clinical trials [41,47].

For future research, our review reveals some gaps. There is a clear need for more longitudinal studies to understand how maternal QoL changes over the course of a child’s development and in response to interventions. It would be particularly valuable to investigate whether treating a child’s ADHD (with medication and/or therapy) leads to measurable improvements in maternal QoL—Guerro-Prado et al. provide encouraging evidence of this in the short term [23], but longer-term studies would help. Additionally, more research is needed in diverse cultural settings. While we included studies from several regions, there are many parts of the world where data on this topic is lacking. Cultural attitudes towards motherhood and disability could modulate QoL outcomes, so understanding those nuances is important for tailoring interventions.

Standardization of QoL measures in this field would also be beneficial. The heterogeneity of instruments used (WHOQOL, EQ-5D, PedsQL, etc.) makes it challenging to compare results directly. The field might benefit from consensus on a core outcome set for parental QoL or caregiver burden in ADHD, which could include both generic QoL metrics and ADHD-specific impact measures. Therefore, future studies should adopt standardized quality-of-life measures or develop a core outcome set for parental well-being in ADHD to enable more direct comparisons across contexts.

Finally, intervention studies targeting maternal QoL should be a priority. It is not enough to solve the problem; we need to test solutions. Programs that combine psychoeducation about ADHD, skills training for behavior management, and components for enhancing maternal well-being (like cognitive–behavioral techniques to reduce stress, or structured social support opportunities) should be evaluated for their effectiveness in improving maternal QoL, as well as any secondary benefits for child outcomes. Early evidence suggests parent-focused psychoeducation and behavioral training can improve parent and family outcomes, but rigorous trials with caregiver-centered QoL endpoints and longer follow-up are still needed [47].

## 5. Conclusions

This scoping review highlights that mothers of children with ADHD are an underserved population facing substantial challenges in their quality of life. The results from eight studies conducted in diverse settings reveal a concerning conclusion: mothers of children with ADHD showed significantly reduced quality of life across multiple dimensions—emotional, social, and daily functioning—compared to mothers of neurotypical children. The strain on these mothers is intensified by the severity of the child’s symptoms, comorbid conditions, maternal mental health issues, and inadequate social support.

These results underscore the necessity of adopting a family-centered approach in the treatment of ADHD. This approach would include successful treatment for the child’s ADHD symptoms with tailored support for the mother. This includes not only regulating the child’s behavior through evidence-based interventions (such as behavioral therapy and appropriate prescription of medications), but also methodically assessing and addressing the mother’s psychological distress, offering resources that strengthen her coping mechanisms and resilience, and fostering social support networks (such as parent support groups or respite care services). Interventions such as parental stress management programs, counseling, and specifically designed parent training and psychoeducation should be incorporated into the care plan for families of children with ADHD.

Improved maternal well-being can significantly affect parenting quality and consistency, potentially resulting in improved outcomes for children with ADHD, including more effective behavior management and a more comfortable home environment. Assisting mothers in coping is intrinsically linked to the treatment of the child, as it is an integral and essential aspect of holistic ADHD management.

In conclusion, our review clarifies that the impact of childhood ADHD extends beyond the child, profoundly influencing mothers within the family unit. Interventions for ADHD must address both the child’s symptoms and the mother’s well-being; only an integrated family-centered approach can reduce the burden of the disorder and improve outcomes for all family members. Future research ought to focus on developing and monitoring interventions targeted at mothers and families, as well as addressing the highlighted gaps, particularly the necessity for longitudinal studies and standardized quality-of-life indicators. By applying a comprehensive, family-centered approach, we can strive to alleviate the impact of ADHD on families and improve the health and well-being of both children and their caregivers.

## Figures and Tables

**Table 1:** Characteristics of the included studies.

Study (Year)	Country	Design	Sample	Comparison Group
Cappe et al. (2017) [21]	France	Cross-sectional survey	*N* = 90 parents (81% mothers); children mean age 9.8; 73% on medication	No direct control (analysis of predictors within ADHD group)
Liang et al. (2021) [7]	Taiwan	Cross-sectional	*N* = 203 mothers; children aged 6–18 (mean ~11)	Yes—mothers of children without ADHD (*n* = 162)
Peasgood et al. (2021) [22]	United Kingdom	Cross-sectional (national online survey)	*N* = 211 parents (90% mothers); children aged 5–15	Yes—parents of children without ADHD (*n* = 211, matched by demographics) *
Piscitello et al. (2022) [8]	USA	Cross-sectional (analysis of cohort data)	*N* = 129 mothers; adolescents aged 13–17	Yes—mothers of adolescents without ADHD (*n* = 118) *
Ahmed et al. (2022) [9]	Saudi Arabia (Middle East)	Cross-sectional comparative	*N* = 108 caregivers (majority mothers) of children with ADHD; children 6–12	Yes—caregivers of children without ADHD (*n* = 108)
Azazy et al. (2018) [13]	Egypt	Cross-sectional clinic-based	*N* = 125 parents (59% mothers); children aged 6–14	No direct control (compared to norms; assessed family function)
Alenezi et al. (2024) [12]	Saudi Arabia	Cross-sectional (online survey)	*N* = 156 parents (60% mothers); children aged 6–17	No direct control (focus on within-group factors; “ripple effect” study)
Guerro-Prado et al. (2016) [23]	Spain (multi-center)	Prospective, longitudinal	*N* = 429 families (71% mothers among respondents); children 6–17, newly diagnosed and starting treatment	No separate control (within-subject pre- vs. post-treatment comparison)

* For the UK and USA studies, the comparison group was drawn from the same survey or dataset, matched or statistically adjusted to mirror the ADHD group’s characteristics.

**Table 2:** QoL assessment tools and summary of main findings in each included study.

Study (Reference)	QoL Measure(s)	Key Outcomes on Maternal QoL	Notable Predictors/Correlates of QoL
Cappe et al. (2017) [21]	Quality of Life in Parents (QoL-P) questionnaire—a parental QoL scale (covering emotional, physical, family, daily activities); plus Perceived Stress Scale and coping measures.	Mothers of ADHD children had low QoL, especially in psychological well-being and daily life activities. High stress levels were reported; many mothers described feeling emotionally drained. QoL scores indicated that ~60% of parents were dissatisfied with their overall QoL.	**Child factors:** ADHD symptom severity (higher hyperactivity/impulsivity associated with lower maternal QoL). **Mother factors:** Emotion-focused coping strategies (e.g., feelings of helplessness, guilt) were linked to poorer QoL. Higher perceived stress and maternal ADHD symptoms were associated with lower QoL. **Social factors:** Low spousal support and marital strain correlated with worse maternal QoL (highlighting the buffering effect of partner support).
Liang et al. (2021) [7]	WHOQOL-BREF (World Health Organization QoL Brief)—assesses Physical, Psychological, Social, Environmental domains. Also used Family APGAR (family support), CAST (child autism traits), SNAP-IV (child ADHD symptoms), CES-D (maternal depression).	Mothers of children with ADHD had significantly worse HRQoL in all 4 WHOQOL domains than mothers of typically developing (TD) children. Notably, 66% of ADHD mothers rated overall QoL “poor” vs. 28% of control mothers. The largest QoL gaps were in psychological and social domains (mothers felt less emotionally stable and less socially supported).	**Child factors:** Inattention severity and presence of autistic traits in the child were strong predictors of lower maternal QoL. Child sleep problems and behavioral issues also predicted poorer QoL. **Mother factors:** Maternal depression was the strongest mother-related predictor of low QoL. **Family factors:** Low perceived family support (APGAR) and low family income were associated with worse QoL. Single mothers had lower QoL than married mothers. In multivariate analysis, maternal and family factors (depression, support) explained more variance in QoL than child clinical factors.
Peasgood et al. (2021) [22]	EQ-5D-5L (EuroQoL 5-Dimensions) for health utility; S-WEMWBS (Short Warwick-Edinburgh Mental Well-being Scale) for mental well-being; questions on life satisfaction, sleep, and time use. Also gathered data on parental work hours, relationships, etc.	Parents (mostly mothers) of ADHD children had significantly lower EQ-5D utility scores (mean ~0.78 vs. ~0.90 in controls, adjusted), indicating worse overall health-related QoL. They also reported poorer sleep quality and less satisfaction with leisure time. Mental well-being scores (WEMWBS) were lower, and life satisfaction was reduced by ~1.2 points (on a 0–10 scale) in the ADHD group. However, there was no significant difference in self-rated physical health or satisfaction with income.	**Child factors:** Greater impact observed when the child had comorbid conditions or more severe ADHD (notably, analyses controlling for child symptom severity still found a residual QoL impact). **Mother factors:** A positive screen for adult ADHD in the parent further worsened QoL. Parental mental health (higher anxiety/depression levels) strongly correlated with QoL. **Contextual factors:** Single parents and those unemployed had lower QoL (the study adjusted for these). Importantly, controlling for maternal ADHD symptoms and socioeconomic status did not fully eliminate the QoL gap, suggesting the child’s ADHD itself imposes a unique burden.
Piscitello et al. (2022) [8]	EuroQol-5D with index values to calculate QALYs (Quality-Adjusted Life Years); also CES-D (maternal depression) and other surveys.	Mothers of adolescents with ADHD had significantly lower health utility scores (mean EQ-5D ≈0.73) than mothers of non-ADHD teens (≈0.93). This equated to a sizable QoL deficit: an average loss of ~0.14 in utility (on 0–1 scale) per year of the child’s life due to ADHD, summing to ~1.96 lost QALYs across ages 5–18. The ADHD-group mothers also reported more problems with anxiety/depression and usual activities, which were the EQ-5D domains most affected.	**Child factors:** The adolescent having an ADHD diagnosis was the strongest predictor of reduced maternal QALY (largest effect among factors). Adolescent behavioral issues (especially disciplinary problems at home) were significantly associated with lower maternal QoL. **Mother factors:** Maternal depression was the only parent characteristic that significantly predicted lower QoL in the final model (mothers with higher CES-D scores had worse utilities). Interestingly, higher maternal education level showed a slight negative association with QoL (more educated mothers reported somewhat lower QoL), which the authors suggested might relate to different expectations or employment stresses.
Ahmed et al. (2022) [9]	WHOQOL-BREF (likely used, given context) or SF-36 domains (not explicitly named in article) for QoL; plus measures of caregiver self-competence and self-liking scales. Also used a Parenting Stress Index.	Caregivers of ADHD children had significantly lower QoL scores across multiple domains compared to caregivers of non-ADHD children (*p* < 0.001 for overall QoL). Approximately two-thirds of ADHD caregivers rated their QoL as poor vs. about one-quarter of controls (consistent with other studies). Notably, ADHD caregivers had much higher parenting stress and worse self-concept (lower self-competence and self-esteem). Many in the ADHD group reported feelings of inadequacy in their parenting role.	**Child factors:** Having a child with ADHD (versus not) was associated with a marked QoL reduction (by study design). Within the ADHD group, those with comorbid disorders or more severe symptoms had caregivers with even lower QoL (though details were not provided, this was implied). **Mother factors:** Lack of social support and higher perceived stress were strongly correlated with poorer QoL. Mothers who reported lower self-efficacy in handling their child’s problems had lower QoL. **Context:** Although not measured quantitatively, the study discussion noted that cultural factors (e.g., stigma in some communities) might influence caregiver well-being.
Azazy et al. (2018) [13]	WHOQOL-BREF for parental QoL; Family APGAR for family function. Also collected demographic and clinical data.	Mothers of children with ADHD had significantly impaired QoL in all WHOQOL domains (scores were lower than population norms) and poor family functioning in 79% of cases. A high proportion of these mothers described their QoL as “bad” or “very bad.” The family APGAR indicated that a majority of families were dysfunctional (low support). QoL was especially low in the psychological domain for mothers, aligning with high stress levels reported.	**Child factors:** The presence of psychiatric comorbidities in the child (e.g., autism, oppositional defiant disorder) was linked to even lower maternal QoL. **Mother factors:** Although Azazy et al. did not directly measure maternal depression/anxiety, it is likely that many mothers had these issues; family dysfunction and maternal distress were correlated. **Family factors:** Family dysfunction (low cohesion/support as per APGAR) was strongly correlated with poor QoL. Additionally, mothers from lower-income households had significantly worse QoL (socioeconomic strain exacerbating stress). In regression analysis, poor family support and low income emerged as significant predictors of low QoL.
Alenezi et al. (2024) [12]	PedsQL Family Impact Module (evaluates parent HRQoL and family functioning) and DASS-21 (Depression Anxiety Stress Scale) for parental mental health. (Also administered an ADHD knowledge and stigma questionnaire in the study).	This study found that parents of children with ADHD had lower family QoL scores and higher rates of mental health issues. About 55% of mothers reported poor family QoL. High parental stress and depression scores were prevalent. Notably, many parents reported experiences of stigma or discrimination related to their child’s ADHD, and this was associated with worse QoL (a unique aspect measured by a stigma questionnaire). Overall, the “ripple effect” of ADHD on parents’ mental health was evident: nearly half of the mothers had moderate to severe depressive symptoms.	**Child factors:** Not detailed in the abstract, but presumably ADHD symptom severity impacted parent QoL (e.g., parents of more severe cases had lower QoL). **Mother factors:** Parental psychological distress (high DASS anxiety/depression) was a key correlate of low QoL. Mothers who felt socially stigmatized or who had less ADHD-related knowledge tended to have worse QoL. **Social factors:** Lower maternal education and being unmarried were mentioned as risk factors for poorer QoL (these often relate to resource availability and support). The authors emphasize the need for psychoeducation and community support to alleviate the burden on parents.
Guerro-Prado et al. (2016) [23]	Family Strain Index (FSI)—measures parental stress and strain on family due to the child’s condition. Child ADHD symptoms measured by Conners scale. (*QoL not directly measured, but stress is an inverse proxy*).	Family stress levels significantly decreased after 2 months of ADHD treatment (methylphenidate) in children. At baseline, parents had very high stress (mean FSI score indicating substantial strain). After 2 months of medication, mean FSI dropped by ~33%, and by 4 months a further slight improvement was seen. There was a strong correlation between reduction in child ADHD symptoms and reduction in parent stress (*r* ≈ 0.8). Families of children with comorbid psychiatric conditions had higher residual stress even after treatment. Although QoL per se was not quantified, the study infers improved parental well-being following effective child treatment.	**Child factors:** Reduction in ADHD symptom severity was tightly linked to improved parent outcomes (each 1-point drop in Conners ADHD score led to a proportionate drop in FSI). However, if the child had comorbid conditions (e.g., anxiety, conduct problems), parents remained more stressed than those whose children had “only” ADHD. **Mother factors:** Not analyzed separately, but 71% of respondents were mothers, and subgroup analysis noted that mothers and fathers showed similar stress reduction patterns. **Family factors:** The authors highlighted that a comprehensive approach (medication + psychosocial support) was recommended to sustain family QoL gains. Families with strong treatment adherence and follow-up had greater improvements in stress.

Note: QoL = quality of life; HRQoL = health-related quality of life; TD = typically developing; QALY = quality-adjusted life year. Higher scores indicate better QoL unless otherwise noted. All differences described were statistically significant (*p* < 0.05). Predictor correlations are drawn from multivariate analyses in the respective studies. Where comparison groups are mentioned, they were included in the study design as indicated.

## Data Availability

No new data were created or analyzed in this study.

## Supplementary Materials

The following supporting information can be downloaded at: https://www.mdpi.com/article/10.3390/children12101376/s1, Table S1: QuADS quality assessment scores for included studies (0 = criterion not met; 3 = fully met).
