# Supplementary material for: Quality of Life in Mothers of Children with ADHD: A Scoping Review
Source: Children (Basel). 2025 Oct 12;12(10):1376. doi: 10.3390/children12101376 (PMC12563816; doi:10.3390/children12101376)
Supplement: Supplementary file 1 [file children-12-01376-s001.zip › children-3898067-supplementary.pdf]

Supplementary File - Quality Appraisal Summary

We examined the methodological quality of each study by using the QuADS tool. The studies had poor quality, characterized by some similar strengths and weaknesses.

As per the strengths, all studies contained well-defined research objectives and employed adequate, validated instruments to measure QoL or related dimensions, thereby meeting essential quality standards. Many of the studies provided comprehensive descriptions of their samples and contexts, with many conducting multivariable analyses to mitigate possible confounders (e.g., controlling for parental mental health or socioeconomic status when assessing the impact of child ADHD) [22]. Comparative studies, conducted with control groups, almost always matched or adjusted for key demographic variables, thus strengthening their results. Inter-rater reliability for diagnoses or consistency in data collection was either reported or suggested in multiple studies such as through the adoption of standardized questionnaires. The prospective study (Guerro-Prado et al.) was multi-centered and had a substantial sample size, hence enhancing its reliability and generalizability [23].

A recurring limitation was the cross-sectional design of almost all of studies, which limits causal inferences (e.g., although maternal depression correlates with diminished QoL, we cannot determine the directionality). Only a single research was longitudinal, and it lacked a non-treatment control; hence, data concerning temporal changes in QoL is limited. Certain studies exhibited possible selection bias; for example, participants in clinic-based research may present with more severe cases or heightened motivation to respond, potentially leading to an overestimation of the burden. Limited research has addressed response bias, indicating that women experiencing heightened distress may be more inclined to engage or provide negative reports. A further issue was the reliance on single-informant reporting, as the same mother frequently assessed both her child's behavior and her own quality of life, so introducing the potential for shared-method variance. Moreover, cultural context may influence perceptions of quality of life; however, each study concentrated on a single country, and only one expressly examined cultural issues such as stigma. Regarding QuADS rating, criteria that were infrequently satisfied included theoretical framing (few research explicitly articulated a guiding theoretical framework for QoL) and reflexivity or bias discussion (about half of the studies comprehensively addressed their limitations or potential biases). None of the cross-sectional research preregistered their analyses or employed numerous informants, for instance. It is important to highlight that scoping reviews do not dismiss studies based on quality; instead, this evaluation informs the interpretation. The convergence of results from studies of differing quality implies robustness; yet, the significant dependence on cross-sectional evidence highlights the necessity for more rigorous future research, such as longitudinal designs.

In conclusion, all studies assessed were deemed to possess at least fair quality, with no significant faults that would undermine their findings. The quality evaluation emphasizes the necessity of cautiously evaluating connections, such as that between maternal sadness and diminished quality of life, with respect to causality. It emphasizes the necessity for further studies to enhance specific methodological features, including the incorporation of longitudinal follow-up, the application of multi-informant data (from both parents or parent and child), and a clear understanding of potential biases in study design.

A QuADS scoring overview for each study has also been developed (Table 3). Each study was assessed based on 13 quality parameters, rated from 0 to 3. The total QuADS scores for the eight studies varied from 21 to 32 out of 39, signifying an overall mediocre quality. Frequently observed high-scoring elements comprised "clear description of aims" and "appropriate methodology," which nearly all studies obtained (scores 2–3). Items with lower scores revealed variability, frequently including "explicit consideration of bias/limitations" and "theory or framework usage," with some studies receiving scores of 1 or 2, suggesting only minimal or moderate attention on these factors. All studies received a minimum grade of 2 for the appropriateness of data analysis, indicating adequate methods for statistical analysis. The QuADS appraisal stresses that although the results are credible, refinements in study design, particularly through longitudinal data and accurate reporting, would strengthen the evidence base.

Table S1. QuADS quality assessment scores for included studies (0 = criterion not met; 3 = fully met).

Study	Clear Aims	Study Design	Context Description	Sample Selection	Data Collection	Data Analysis	Ethics & Bias	Findings	Transferability	Use of Theory	Conclusions	Overall Strengths & Limitations	Total Score (/39)
Cappe et al. (2017) [21]	3	3	3	2	3	3	2	3	2	2	3	2	31
Liang et al. (2021) [7]	3	3	3	3	3	3	2	3	3	1	3	2	32
Peasgood et al. (2021) [22]	3	3	3	3	3	3	2	3	3	1	3	2	32
Piscitello et al. (2022) [8]	3	3	3	2	3	3	2	3	2	1	3	2	30
Ahmed et al. (2022) [9]	3	2	2	2	2	2	1	2	2	0	2	2	22
Azazy et al. (2018) [11]	3	2	2	2	2	2	1	2	2	0	2	1	21
Alenezi et al. (2024) [13]	3	3	3	3	3	3	2	3	2	1	3	2	31
Guerro-Prado et al. (2016) [23]	3	3	3	3	3	3	2	3	3	0	3	2	31

Note: Criteria are abbreviated. Examples: Study Design (appropriate design for aims), Ethics & Bias (ethical considerations, efforts to minimize bias), Transferability (generalizability of findings). All studies were published in peer-reviewed journals and underwent ethics approval. Total scores are approximate, as some subjective judgment is involved in scoring each item.
